# Impact metrics and science evaluation

**DOI:** 10.17843/rpmesp.2022.392.11171

**Published:** 2022-06-30

**Authors:** Lilian N. Calò

**Affiliations:** 1 BIREME/OPS/OMS BIREME/OPS/OMS

**Keywords:** Scientific Communication and Diffusion, Health Research Evaluation, Bibliometric Indicators, Scientific Publication Indicators, Journal Impact Factor, Journals as Topic

## Abstract

Scientists have always looked for ways to evaluate research results to recognize and reward their efforts, and to support decisions regarding programs and public policies. The metrics of scientific impact have become, in recent decades, the driving force behind the academic environment. The work of researchers, scientific journals, databases and publishers, research institutions, and funding agencies is driven by the impact of the research they produce, publish, index, promote and finance. Bibliometric indicators are widely used for the evaluation of scientific output, despite the lack of a clear relationship between citations and quality, impact, or scientific merit. Furthermore, the relationship is even less evident regarding innovation, which is an inherent characteristic of scientific research. This article describes the main types of metrics used to evaluate scientific output, as well as its features, potentials, and limitations.

## INTRODUCTION

The evaluation of science involves the systematic assessment of merit based on the time and the financial and human resources used to achieve an objective. The research evaluation process, which consists of data analysis and reporting, is a rigorous and systematic process that involves the collection of data on organizations, processes, projects, services, and resources. Research evaluation aims to improve decision making and lead to practical applications ^1^.

Therefore, scientific research results should be evaluated in order to determine what is relevant and what is not, as well as to support decisions on project funding and translate scientific output into programs and public policies for society as a whole ^2^.

Buckeridge ^3^ proposes a definition of scientific impact based on the idea of physical impact. “Impact is the capacity of a discovery or a set of discoveries to change the structure of the interaction networks between the ways of thinking of human beings and local or global societies, causing deformations (changes) in the physical world. Impact on the intellectual field causes this disorder in the brain of one or several people. A new idea interferes in the way our brain understands and interprets phenomena”. 

Impact metrics in science have become, in recent decades, the driving force in the academic environment. The work of researchers, scientific journals, databases and publishing houses, research institutions and funding agencies is ruled by the impact of the research they produce, publish, index, promote and fund. 

However, some authors vehemently disagree with the idea of metrics and evaluation of science to map the contributions of innovation to society: “The notion that contributions to the improvement of society by technological or social innovation can always be mapped and measured is erroneous. Likewise, the notion that the main or only purpose of universities is to drive economic growth through innovation, in ways that can be measured with quantitative indicators, is flawed. Science has, quite evidently, contributed immensely to the modernization of society and the vast improvements of living standards in Europe and North America in the past two hundred years, including the development of an economy and a society with less harmful impact on health and the environment. It is time to stop evaluating it with metrics that obviously fail to make justice to its success, and most of all time to stop governing it on basis of what these metrics show. Either Lord Kelvin (or Peter Drucker, or whoever really said it) was wrong in stating that ‘if you can’t measure it, you can’t improve it’, or science does not need improving, or alternative and more accurate means of science evaluation need to be developed. Or maybe all three.” ^4^.

However, academic institutions are conditioned to operate under a series of evaluation metrics that rule career policies, such as hiring, promotion, awards and distinctions, in addition to obtaining financial resources for research, all of which influence the market for publishers and scientific journals, and feed the crowded university rankings.

This article describes the main types of metrics used to evaluate scientific output, their characteristics, potentials and limitations.

## CITATION-BASED METRICS

Bibliometric indicators are widely used for the evaluation of scientific output, despite the lack of a clear relationship between citations and quality, impact, or scientific merit. Furthermore, the relationship is even less evident regarding innovation, which is an inherent characteristic of scientific research ^5^. In addition, there are studies that analyze the complexities of citation ^6^ ^-^ ^8^, which demonstrate how little can be assumed about the true motives to cite the final article. All of this has an impact on the attribution of relevance to articles based exclusively on the citations received and, consequently, on the models of science evaluation overall.

The first known bibliometric indicator is the impact factor (IF®), created in 1972 by Eugene Garfield ^9^ to evaluate journals, with the publication of the Science Citation Index of the Institute for Scientific Information (ISI).

To calculate the IF, the number of citations received by the journal in a given time frame (three or five years) is divided by the number of articles published in that same period. The Web of Science (WoS) database (which belongs to Clarivate Analytics since 2016) is used to count the citations, therefore, citations from the approximately 13,000 journals indexed in this database to date are counted.

Some considerations on the IF calculation should be noted. The IF is an average value per journal and not per article. Furthermore, there are published texts that are not counted as articles (the denominator of the quotient), but citations to these texts can be counted (the numerator). Therefore, it is known that there are artifices that are used by editors to increase the IF of journals. In addition, the database that provides access to the IF of journals, the Journal Citation Reports (JCR), an integral part of the WoS, is accessible by subscription.

The IF remained the main (and only) journal impact index since its creation by Garfield in 1972 until 2008, when the SCImago Journal Rank (SJR), measured in Elsevier’s Scopus database, was launched. The point about the IF is that it was used more than an index to rank journals. Since it is easy to calculate, its use to evaluate researchers, institutions, graduate programs and any other evaluation of scientific production that could benefit from a qualitative or broader evaluation was often reduced to a list of publications associated with an IF.

In 2012, a group of editors and publishers of academic journals gathered at the Annual Meeting of the American Society of Cell Biology in San Francisco, USA, wrote a document that became known as the San Francisco Declaration on Research Assessment ^10^, which recommends that citation-based metrics, such as the IF, should not be used to evaluate researchers in hiring, promotion, or research funding decisions. Currently (April 2022), more than 21,000 people from 158 countries have signed the San Francisco Declaration.

Since 2014, the Leiden Manifesto ^11^, which originated at the 19th International Conference on Science and Technology Indicators in Leiden, The Netherlands, guides the use of science assessment metrics in Europe. The Manifesto has been translated into 25 languages, adopted by institutions and recognized even by publishers worldwide.

In 2004, the multinational publisher Elsevier launched the Scopus database, available online by subscription. In 2007, Spanish researcher Felix Moya-Anegon launched the SCImago Journal Rank (SJR), an impact index created as an alternative to the IF. It is calculated in a similar way to the IF, i.e., citations per article, and is also an average indicator per journal, with the difference that the calculation reflects the prestige of the journal ^12^. For this purpose, the PageRank algorithm is used, which is the same as the one used by Google to list the most visited pages in a search. In addition, it is a size independent indicator and its values rank the journals by the “average prestige per article”. Although Scopus is a subject access database, SJR ^13^ is available in open access.

In the following years, Scopus launched new indexes for the Elsevier family of indicators: Source Normalized Impact per Paper (SNIP), CiteScore metrics and the h-index for journals, which have different characteristics and applications, as described by Elsevier ^14^.

In response to Elsevier’s releases, WoS launched in 2007 the Eigenfactor® and Article Influence® indexes, developed by Carl Bergstrom and Jevin Westen at the University of Washington ^15^. Both indexes use Google’s PageRank algorithm and also take into account the importance of citations received (according to the prestige of the citing journal). Eigenfactor and Article Influence are adjusted for different citation patterns, allowing comparison of the performance of journals from different disciplines and eliminating self-citations. The indexes are independent of their numerical values, unlike the IF. In addition to being available on the JCR website (subscription access), both indexes are available on an open access page ^15^; evidently, only journals included in the JCR have Eigenfactor and Article Influence values attributed to them. It is noteworthy, however, that the precise and extremely elegant calculation of these indexes has not been used in journal evaluation systems of any institution, university ranking or graduate program. Their complexity may seem difficult for users to interpret, even if it allows for more precise analyses.

In 2005, the physicist J.E. Hirsch devised a method ^16^ to quantify the scientific productivity of a researcher, institution, or journal. The h-index is defined as the number of publications with a number of citations ≥ h. Hirsch argues that his index is preferable to other single-number criteria commonly used to evaluate a researcher’s scientific output. The h-index favors researchers with greater scientific seniority, so to allow comparisons between scientists of different ages it is preferable to use the h5 or h10 index. In these cases, publications (and citations) form the last 5 or 10 years are counted.

There are several ways to obtain the h-index of a researcher. In WoS, through the Citation Report resource, or in Google Scholar, through the author’s profile. Usually, the h-index calculated by Google Scholar is higher than in WoS, which only counts the publications indexed in that database.

Digital Science’s (DS) Dimensions research database ^17^, was launched in 2016, for search and query. In 2018, DS relaunched an extended version of Dimensions, a commercial academic search platform that allows searching for publications, datasets, grants, patents, and clinical trials. The free version of the platform only allows searching for publications and datasets. Studies published in 2021 have concluded that Dimensions provides broader temporal and publication source coverage than Scopus and WoS in most subject areas, and that it is closer to Google Scholar in its coverage.

One of the main differences of Dimensions bibliometric indexes compared to WoS and Scopus is that it presents metrics related to the documents and not related to the journals, like the FI and SJR indexes. The metrics presented in the Dimensions Badge refer to citations received by the articles.

## USAGE AND DOWNLOAD METRICS

One of the main challenges in using download and usage indicators to measure the impact of articles, as an alternative to citations or mentions on the web (Altmetric, alternative metric), is the multiple publishers’ platforms where articles are available and the difficulty in adding article download counts to view the total number. 

In order to use download counts as a measure of “impact”, user views of the full text article (HTML) or the PDF downloads are assumed as an indicator of reader interest in the article and, as a consequence, a measure of impact.

The time intervals are one of the advantages of using download measures over citations. While citations are counted at intervals of 2 to 5 years, it is possible to start counting downloads after online publication and obtain consistent indicators after only a few months.

The analysis of usage and download metrics can be very useful for monitoring the performance of journals indexed in databases. For example, it is possible to evaluate, from one year to the next, if the number of downloaded articles of a journal increased or decreased; this data can be compared with the received citations or the trend of the Altmetric index. 

It is important to follow standards of good practice when registering the usage and downloading of articles. The COUNTER Code of Practice ^18^ enables content providers to produce consistent, comparable and reliable usage data for their online content. According to the COUNTER standards, robots and duplicate records are excluded when a user accesses, in the same section, the same article several times, e.g., the user accesses the abstract, then the HTML, then downloads the PDF of the same article.

According to Kurtz and Bollen, ^19^ “Considerable challenges still exist with regard to the standardization of recording and aggregation of usage data. In the present situation usage data are recorded in a plethora of different formats, each representing a different permutation of recording interfaces, data fields, data semantics, and data normalization.”

Therefore, metrics of article usage and downloads cannot be viewed in isolation. Thus, they must be analyzed by comparing, for example, journals in the same area of knowledge, individual articles compared to others, influence of language or year of publication, etc. The closest to the ideal situation occurs when analyzing journals from the same platform, or from a given Publisher, as this eliminates many of the variables listed by Kurtz and Bollen.

For example, the SciELO platform ^20^ provides users with usage data for more than 1400 journals. Using the SciELO Sushi API tool, it is possible to obtain access reports for a particular article, journal or collection. The obtained results can be observed by country of access, year of publication, or language of the document, among others; it is also possible to use parameters to choose the period to be analyzed.

## SOCIAL NETWORKS AS A MEASURE OF SCIENTIFIC IMPACT

Social networks offer new possibilities for scientific communication, creating forms of content dissemination that accelerate the publication and evaluation process, connecting researchers, editors, students, academic institutions, funding agencies and society in general.

One study shows that “less than half of the published scientific articles are cited one or more times, i.e., when we discuss citation as a reference for the use of the article, we inevitably leave out at least half of the research being done in the world” ^21^. This does not mean that the impact of these articles on the scientific community is null, on the contrary. Publications are read, downloaded, shared and cited through social networks, blogs, news channels, public policy and other forms of online presence, collected and measured in indexes such as Altmetric.

Altmetric is a paid service provided by Digital Science for groups of journals or individual journals, which measures the impact of an article based on its dissemination in social networks. This indicator is updated daily, and attributes different scores to each communication channel ^22^.

The speed with which newly published articles are shared on the web is one of the strengths of altmetrics compared to citation-based metrics, which are counted two to three years after publication. In addition, studies indicate that articles with a high social media presence are more widely disseminated and receive more citations. However, it is important to consider the presence of non-English articles in indexes such as Altmetric. Recent studies ^23^ show that out of 140,000 articles published between 2015 and 2018 in Latin American and Caribbean journals in Portuguese, Spanish and English, only 13% were mentioned on the social web. Of this fraction, 57% of the mentions were for articles in English, 24% in Spanish and 18% in Portuguese.

It should also be considered that most of the developments and web applications, especially those academic, are created by researchers for publications in English, this could result in bias in the monitoring of publications in non-English-speaking nations.

## FINAL CONSIDERATIONS

Metrics for research evaluation evolve, change, new methodologies emerge and ways to improve existing methods are discussed. One topic we did not address in this article is the evaluation of research projects, not because it is not important, on the contrary, it is quite important, but it would be an even more extensive discussion. Research institutions and funding agencies around the world are discussing what is the most efficient way to conduct peer review of grant proposals ^24^, like whether it is valid to open the evaluation, as is being done with the review of articles, according to open science practices. In any case, it is a very simple issue, because if an article is rejected for publication, it can influence the career of a researcher. The evaluation of a project, however, has a more direct influence in an area of knowledge, because if the project is not funded, it may never be carried out. The metrics for evaluating science must be considered very seriously, in order not to stop science itself.
