# Supplementary material for: Impact metrics and science evaluation
Source: Rev Peru Med Exp Salud Publica. 2022 Jun 30;39(2):236–40. doi: 10.17843/rpmesp.2022.392.11171 (PMC11397581; doi:10.17843/rpmesp.2022.392.11171)
Supplement: Translation

# Translation: Métricas de impacto y evaluación de la ciencia

**Authors:** Lilian N. Calò

**Affiliations:** 1 BIREME/OPS/WHO

**Keywords:** Comunicación y Divulgación Científica, Evaluación de la Investigación en Salud, Indicadores Bibliométricos, Indicadores de Producción Científica, Factor de Impacto de Revistas, Revistas como Tema

## Abstract

Los científicos siempre buscaron formas de evaluar los resultados de las investigaciones para reconocer y premiar sus esfuerzos, y para apoyar decisiones en programas y políticas públicas. Las medidas o métricas de impacto de la ciencia se transformaron, en las últimas décadas, en el motor propulsor del ambiente académico. Investigadores, revistas científicas, bases de datos y casas editoriales, instituciones de investigación y agencias de financiación tienen su trabajo gobernado por el impacto de la investigación que producen, publican, indexan, promueven y financian. Se utilizan largamente indicadores bibliométricos para la evaluación de la producción científica, a pesar de la falta de relación inequívoca entre citas y calidad, impacto o mérito científico. Además, cuando se considera la innovación, característica inherente de la investigación científica, la relación es todavía más desconectada. En este artículo se describen los tipos principales de métricas utilizadas para evaluar la producción científica, sus características, potenciales y limitaciones.

## INTRODUCCIÓN

La evaluación de la ciencia se refiere a la valoración sistemática del mérito en función del tiempo y los recursos financieros y humanos empleados para lograr un objetivo. El proceso de evaluación de la investigación, que consiste en el análisis de datos e informes, es un proceso riguroso y sistemático que implica la recopilación de datos sobre organizaciones, procesos, proyectos, servicios y recursos. La evaluación de la investigación tiene por objetivo mejorar la toma de decisiones y conducir a aplicaciones prácticas ^1^.

Se pretende, entonces, evaluar los resultados de la investigación científica para saber lo que es relevante y lo que no para apoyar las decisiones sobre financiación de proyectos y para traducir esta producción científica en programas y políticas públicas para toda la sociedad ^2^.

Buckeridge ^3^ propone una definición de impacto científico basado en la idea del impacto físico: «Impacto es la capacidad que tiene un descubrimiento o un conjunto de descubrimientos en cambiar la estructura de las redes de interacción entre las formas de pensar de los seres humanos y sociedades locales o globales, generando deformaciones (cambios) en el mundo físico. El impacto en el campo intelectual provoca este *desorden* en el cerebro de una o varias personas. Una nueva idea interfiere en la forma con que nuestro cerebro entiende e interpreta los fenómenos». 

Las medidas o métricas de impacto de la ciencia se transformaron, en las últimas décadas, en el motor propulsor del ambiente académico. Investigadores, revistas científicas, bases de datos y casas editoriales, instituciones de investigación y agencias de financiación tienen su trabajo gobernado por el impacto de la investigación que producen, publican, indexan, promueven y financian. 

Hay autores, sin embargo, que disienten vehementemente de la idea de métricas y evaluación de la ciencia para mapear las contribuciones de la innovación a la sociedad: «La noción de que las contribuciones a la mejora de la sociedad por medio de la innovación tecnológica o social siempre pueden mapearse y medirse es errónea. Asimismo, la noción de que el propósito principal o único de las universidades es impulsar el crecimiento económico a través de la innovación, en formas que puedan medirse con indicadores cuantitativos, es errónea. Evidentemente, la ciencia ha contribuido enormemente a la modernización de la sociedad y a las grandes mejoras de los niveles de vida en Europa y América del Norte en los últimos doscientos años, incluido el desarrollo de una economía y una sociedad con un impacto menos dañino en la salud y el medioambiente. Es hora de dejar de evaluarla con métricas que, obviamente, no logran hacer justicia a su éxito y, sobre todo, de dejar de gobernarla sobre la base de lo que muestran estas métricas (...) o quienes afirma que “si no puedes medir, no puedes mejorar” está equivocado, o la ciencia no necesita mejorar, o es necesario desarrollar medios alternativos más precisos de evaluación de la ciencia. O tal vez, todos los tres» ^4^ (traducción libre del original en inglés).

No obstante, las instituciones académicas están condicionadas a operar bajo una serie de métricas de evaluación que gobiernan políticas de carrera, como contratación, promoción, premios y distinciones, además de la obtención de recursos financieros para la investigación, influencian el mercado de editoriales y revistas científicas, y alimentan los concurridos *rankings* universitarios.

En este artículo se describen los tipos principales de métricas utilizadas para evaluar la producción científica, sus características, potenciales y limitaciones.

## MÉTRICAS BASADAS EN CITAS

Se utilizan largamente indicadores bibliométricos para la evaluación de la producción científica, a pesar de la falta de relación inequívoca entre citas y calidad, impacto o mérito científico. Además, cuando se considera la innovación, característica inherente de la investigación científica, la relación es todavía más desconectada ^5^. Existen estudios que analizan las complejidades de la práctica de citación ^6^ ^-^ ^8^, y demuestran lo poco que se puede suponer sobre los verdaderos motivos para citar el artículo final. Todo eso tiene impacto principalmente en la atribución de relevancia a los artículos en base exclusivamente de las citas recibidas y, por consiguiente, en los modelos de evaluación de la ciencia en general.

El primer indicador bibliométrico que se tiene noticia es el factor de impacto (FI®), creado en 1972 por Eugene Garfield ^9^ para evaluar revistas, con la publicación del Science Citation Index del Institute for Scientific Information (ISI). 

Para calcular el FI, se divide el número de citas recibidas por la revista en un determinado intervalo de tiempo (tres o cinco años) por el número de artículos publicados en ese mismo intervalo. Para contabilizar las citas, se utiliza la base de datos de Web of Science (WoS), (que desde el 2016 pertenece a Clarivate Analytics), y así se cuentan solo las citas provenientes de revistas indexadas en esta base de datos, que incluye, a la fecha, aproximadamente 13 000 revistas.

Algunas consideraciones sobre el cálculo del FI deben ser observadas. Se trata de un valor promedio por revista y no por artículo. Además, hay textos que se publican en la revista que no se cuentan como artículos (el denominador del cociente), pero las citas a estos mismos textos se pueden contabilizar (el numerador). Por eso, es sabido que existen artificios que son utilizados por los editores para aumentar el FI de revistas. Además, la base de datos que da acceso a los FI de las revistas, el Journal Citation Reports (JCR), parte integrante de la WoS, es accesible a través de subscripción. 

El FI permaneció como el principal (y único) índice de impacto de revistas desde su creación por Garfield, en 1972, hasta el 2008, cuando se lanzó el SCImago Journal Rank (SJR), medido en la base de datos Scopus, de Elsevier. La cuestión sobre el FI es que se utilizó más allá de un índice para ranquear revistas. Por ser un índice fácil de calcular, su utilización para evaluar investigadores, instituciones, programas de posgrado y cualquier otra forma de medir la producción científica que pudiera beneficiarse de una evaluación cualitativa o de carácter más amplio, fue muchas veces reducida a una lista de publicaciones asociadas a un FI.

En 2012, un grupo de editores y editoriales de revistas académicas reunidos en el Annual Meeting of the American Society of Cell Biology en San Francisco, EE. UU., escriben un documento que luego fue conocido como la San Francisco Declaration on Research Assessment ^10^, la cual recomienda que no se utilicen métricas basadas en citas, como el FI, para evaluar a investigadores en situación de contratación, promoción, o en decisiones de conceder financiamiento para proyectos de investigación. En la actualidad (abril de 2022), más de 21 000 personas de 158 países firmaron esta declaración.

En 2014, el Manifiesto de Leiden ^11^, originado en la 19.° Conferencia Internacional Sobre Indicadores en Ciencia y Tecnología, en Leiden, Holanda, orienta el uso de métricas de evaluación de la ciencia en Europa. Este documento ha sido traducido a 25 idiomas, adoptado por instituciones y reconocido por editoriales de todo el mundo. 

En 2004, la editorial multinacional Elsevier lanza la base de datos Scopus, disponible en línea por subscripción. En 2007, el investigador español Félix Moya-Anegón lanza el SCImago Journal Rank (SJR), un índice de impacto creado como una alternativa al FI. Se calcula de forma análoga al FI, o sea, citas por artículo, y es también un indicador promedio por revista, con la diferencia que el cálculo refleja el prestigio de la revista ^12^. Para eso, se utiliza el algoritmo PageRank, el mismo de Google para ordenar las páginas más visitadas en una búsqueda. Además, es un indicador independiente de tamaño y sus valores ordenan las revistas por el «prestigio medio por artículo». A pesar de que Scopus es una base de acceso por asignatura, SJR ^13^ está disponible en acceso abierto.

En los años siguientes, Scopus lanza nuevos índices para la familia de indicadores de Elsevier: *Source Normalized Impact per Paper* (SNIP), *CiteScore metrics* y el índice h para las revistas, que tienen características y aplicaciones distintas, según descripción de Elsevier ^14^.

Para responder a los lanzamientos de Elsevier, WoS lanza en 2007 los índices Eigenfactor® y Article Influence®, desarrollados por Carl Bergstrom y Jevin Westen en la Universidad de Washington ^15^. Ambos índices utilizan el algoritmo PageRank de Google y también tienen en cuenta la importancia de las citas recibidas (según el prestigio de la revista que cita). Eigenfactor y Article Influence se ajustan para diferentes padrones de citas, permitiendo comparar desempeños de revistas de distintas disciplinas y eliminan las autocitaciones. Los índices son independientes de sus valores numéricos, al contrario del FI. Además de estar disponibles en la página web del JCR (acceso por subscripción), los dos índices están disponibles en una página de acceso abierto ^15^; evidentemente, apenas las revistas incluidas en el JCR tienen valores de Eigenfactor y Article Influence atribuidos. Es notable, que el cálculo preciso y extremamente elegante de estos índices no se utilicen en sistemas de evaluación de revistas de alguna institución, *rankings *universitarios o programas de posgrado. Su complejidad puede parecer difícil de interpretar, aunque permita análisis más precisos.

En 2005, el físico J.E. Hirsch idealizó un cálculo ^16^ para cuantificar la productividad científica de un investigador, institución o revista. El índice h es definido como el número de publicaciones con número de citas ≥ h. Hirsch argumenta que su índice es preferible a otros criterios de un solo número comúnmente utilizados para evaluar la producción científica de un investigador. Como el índice h favorece a investigadores con mayor antigüedad científica, para permitir comparaciones entre científicos de distintas edades, es preferible utilizar el índice h5 o h10. En esos casos se cuentan las publicaciones (y las citas) en los últimos 5 o 10 años.

Existen varias formas de obtener el índice h de un investigador. En la WoS, a través del recurso Citation Report, o en Google Scholar, por medio del perfil del autor. En general, el índice h calculado por Google Scholar es mayor que en la WoS, que solo cuenta las publicaciones indexadas en esa base de datos.

La base de datos de investigación Dimensions ^17^ de Digital Science (DS), se lanzó en 2016, para búsqueda y consulta. En 2018, DS relanzó una versión extendida de Dimensions, una plataforma comercial de búsqueda académica que permite buscar publicaciones, conjuntos de datos, subsidios, patentes y ensayos clínicos. La versión gratuita de la plataforma solo permite buscar publicaciones y conjuntos de datos. Estudios publicados en 2021 han concluido que Dimensions proporciona una cobertura temporal y de fuente de publicación más amplia que Scopus y WoS en la mayoría de las áreas temáticas, y que es más cercana a Google Scholar en su cobertura. 

Una de las principales diferencias de los índices bibliométricos de Dimensions comparado a WoS y Scopus es que presenta métricas relativas a los documentos y no a las revistas, como los índices FI y SJR. Las métricas presentadas en el Dimensions Badge se refieren a citas recibidas por los artículos.

## MEDIDAS DE USO Y DESCARGA

Uno de los principales desafíos en la utilización de indicadores de uso y descarga para medir impacto de artículos, como alternativa para citaciones o mención en la web (*Altmetric, alternative metric*), es la multiplicidad de plataformas de las editoriales en que los artículos están disponibles y la dificultad en agregar las cuentas de descargas de artículos para obtener una visión del total.

Para utilizar las medidas de descarga como índice de «impacto» de un artículo, se presupone que cuando un usuario visualiza el texto completo (HTML) de un artículo o descarga el PDF, se trata de una indicación de interés del lector por el artículo y como consecuencia, una medida de impacto.

Una de las ventajas de utilizar medidas de descarga sobre las citaciones reside en los intervalos de tiempo. Mientras las citas se cuentan en intervalos de 2 a 5 años, es posible empezar a contar descargas luego de la publicación en línea y obtener indicadores consistentes después de solo algunos meses. 

El análisis de uso y descarga pueden ser muy útiles para acompañar el desempeño de revistas indexadas en bases de datos. Por ejemplo, se puede evaluar de un año al otro si una revista registró un aumento o disminución de descargas de sus artículos, y comparar esos datos con citas recibidas o la tendencia del índice *Altmetric* de sus artículos.

Es importante adoptar normas de buenas prácticas al usar y descargar artículos. El Código de Practicas COUNTER ^18^ permite a los proveedores de contenido producir datos de uso consistentes, comparables y creíbles para su contenido en línea. De acuerdo con las normas COUNTER, se excluyen robots y registros duplicados cuando un usuario accede, en una misma sección, varias veces al mismo artículo, por ejemplo, accede al resumen, luego al HTML y luego descarga el PDF de un mismo artículo.

Según Kurtz y Bollen ^19^ «Todavía existen desafíos considerables con respecto a la estandarización de registro y agregación de datos de uso. En la situación actual, los datos de uso se registran en una plétora de formatos diferentes, cada uno de los cuales representa una permutación diferente de interfaces de registro, campos de datos, semántica de datos y normalización de datos». 

Por eso, las medidas de uso y descarga de artículos no son grandezas que tienen un significado aislado. Así se analizan por comparación entre, por ejemplo, revistas de la misma área del conocimiento, artículos individuales comparados a otros, influencia del idioma o del año de la publicación, etc. La situación más próxima al ideal se presenta cuando se analizan revistas de una misma plataforma, o de una determinada editorial, pues así se eliminan muchas de las variables enumeradas por Kurtz y Bollen.

Por ejemplo, la plataforma SciELO ^20^ proporciona a los usuarios datos de uso de más de 1400 revistas. Mediante la herramienta denominada SciELO Sushi API es posible obtener informes de acceso para un artículo, revista o colección en particular. Los resultados obtenidos se pueden observar por país de origen de las personas que accedieron, año de publicación de los documentos, o idioma del documento, entre otros; es posible, además, informar parámetros para elegir el período a analizar.

## REDES SOCIALES COMO MEDIDA DE IMPACTO CIENTÍFICO

Las redes sociales ofrecen nuevas posibilidades en la comunicación científica, creando formas de diseminación de contenidos que aceleran el proceso de publicación y evaluación, conectando investigadores, editores, estudiantes, instituciones académicas, agencias financiadoras y la sociedad en general.

Un estudio muestra que «menos de la mitad de los artículos científicos publicados se citan una o más veces, es decir, cuando discutimos de citación como referencia para el uso del artículo, invariablemente dejamos de lado, al menos, la mitad de la investigación que se hace en el mundo» ^21^. Eso no significa que el impacto de esos artículos en la comunidad científica sea nulo, al contrario. Las publicaciones se leen, descargan, comparten y se citan a través de las redes sociales, blogs, canales de noticias, políticas públicas y otras formas de presencia en línea, recopilados y medidos en índices como *Altmetric*.

*Altmetric* es un servicio pagado proporcionado por Digital Science para grupos de revistas o revistas individuales, e indica el impacto de un artículo con base en su diseminación en redes sociales. Este indicador se actualiza diariamente, y atribuye diferentes puntajes a cada canal de comunicación ^22^.

La velocidad con la que los artículos recién publicados se comparten en la web es uno de los puntos fuertes de las altmetrías en comparación con las métricas basadas en citas, que se cuentan dos o tres años después de la publicación. Además, los estudios indican que los artículos con alta presencia en las redes sociales son más difundidos y reciben más citas. Sin embargo, es importante hacer una consideración sobre la presencia de artículos en idioma diferente del inglés en índices como *Altmetric*. Estudios recientes ^23^ muestran que de 140 mil artículos publicados entre 2015 y 2018 en revistas de América Latina y el Caribe en portugués, español e inglés, solo el 13% fueron mencionados en la web social. De esta fracción, el 57% de las menciones era para artículos en inglés, 24% en español y 18% en portugués. 

Se debe considerar que la mayoría de los desarrollos y aplicaciones para la web, especialmente los de aplicación académica, son elaborados por investigadores para publicaciones en idioma inglés, esto produciría sesgos en el seguimiento de las publicaciones en las naciones no angloparlantes.

## CONSIDERACIONES FINALES

Las métricas para la evaluación de la investigación evolucionan, cambian, surgen nuevas metodologías y se discuten formas de perfeccionar mecanismos existentes. Un tema que no abordamos en este artículo es la evaluación de proyectos de investigación, y no porque no sea importante, al contrario, es muy importante, pero esto tomaría muchas páginas. Instituciones de investigación y agencias de financiación en todo el mundo discuten sobre cuál es la forma más eficiente de conducir la evaluación por pares de las propuestas de subsidio ^24^, si es válido abrir la evaluación, como se empieza a hacer con la revisión de artículos, según las prácticas de ciencia abierta. De cualquier forma, es un tema muy sencillo, pues si un artículo es rechazado para publicación, puede influenciar la carrera de un investigador. La evaluación de un proyecto, sin embargo, tiene influencia más directa en un área del conocimiento, pues si la investigación no se financia, es posible que nunca llegue a buen término. Se debe considerar muy seriamente las métricas para evaluar la ciencia, con el fin de no detener la ciencia misma.
